# Editorial: Novel advances in gastrointestinal cancer treatment

**DOI:** 10.3389/fmolb.2023.1238098

**Published:** 2023-06-30

**Authors:** Yanqing Liu, Shicheng Guo, Tao Yuan, Yang Chen

**Affiliations:** ^1^ Herbert Irving Comprehensive Cancer Center, Columbia University, New York, NY, United States; ^2^ Department of Medical Genetics, School of Medicine and Public Health, University of Wisconsin-Madison, Madison, WI, United States; ^3^ College of Life Sciences, Jiangxi Normal University, Nanchang, China; ^4^ CAS Key Laboratory of Separation Science for Analytical Chemistry, Dalian Institute of Chemical Physics, Chinese Academy of Sciences, Dalian, China

**Keywords:** gastrointestinal cancer, tumor therapy, microbiota, senescence, non-coding RNA, microRNA, prognostic model

There are exciting times ahead for exploring the biological mechanisms of gastrointestinal cancer and discovering new drugs for therapy, but there are also unsatisfied needs that aim at both understanding specifically gastrointestinal cancer and discovering novel drug targets for drug development in this type of cancer. Over the past years, tremendous advances and successes have been made in progressive principle and technique for current gastrointestinal cancer therapy, including anti-PD-1/PD-L inhibitors, antibody drug conjugates (ADC) and aptamers, as well as more recent chimeric antigen receptor T-cell (CART) therapy, proteolysis targeting chimeric (PROTAC), RNA therapy (siRNA and mRNA therapy), gene therapy and their combination in gastrointestinal cancer patients. However, there are still many challengers in gastrointestinal cancer cures for most patients because of individual differences, drug resistance and reoccurrence. Identification of the mechanisms and therapeutic targets involved in gastrointestinal cancer has proven to be a promising field, which would be helpful not only for the prevention, but also to improve the understanding and characterization of gastrointestinal cancer, and to optimize and personalize the treatment. Multidisciplinary research has been performed to explore new opportunities in treatment that may increase the overall survival of patients, however, the side effects such as cytotoxicity and development of drug resistance remain and have limited the clinical practice. The specific mechanisms of gastrointestinal cancer and promising drug targets need to be further identified, which will provide more reliable data for precise treatment and improve the clinical outcomes of cancer.

To collect novel scientific findings and strategies in gastrointestinal cancer biological mechanisms and drug discovery, and to improve future treatments, we organized this Research Topic entitled “*Novel advances in gastrointestinal cancer treatment*.” Six papers (including four reviews, one research article, and one case report) have been compiled in this Research Topic. Here we would like to briefly introduce these papers.

In recent years, microbiota in human gut tract is a hot spot in research. Microbes in the gut not just simply colonize there. Instead, they have profound influences on the physiopathology of the gut (Hou et al., 2022). Particularly, gut microbiota has been demonstrated to be involved in the initiation and progression of colorectal cancer, and influence the outcome of colorectal cancer treatment (Cheng et al., 2020). Li et al. introduced the effects of gut microbiota on the colorectal cancer microenvironment, with a focus on the immune cells in it. Mechanistically, gut microbiota exerts these functions mainly by secreting diverse metabolites, including short-chain fatty acids, bile acids, inosine, and polyamines. These effects together contribute to the development of colorectal cancer and also provide new avenues to treat it. Based on this, authors then discussed how traditional Chinese medicine can be used to treat colorectal cancer by regulating the gut microbiota.

Liu et al. here presented a good review focusing on cell senescence in gastrointestinal cancer. For a single normal cell, senescence means permanent arrest in cell cycle. Eventually, this cell will die without proliferation. However, for a tumor as a cell community, the happening of tumor cell senescence may have controversial effects on tumor development (Collado and Serrano, 2010). To clarify this issue, the authors differentiated two types of cell senescence: cell-autonomous senescence and cell non-autonomous senescence. They depicted the complex role of cell senescence in tumor therapy. In addition, they also introduced the mechanism and biomarker of senescence, and the therapeutic opportunity in gastrointestinal cancer by targeting senescent cells.

Non-coding RNA is composed of many types of RNAs, such as microRNA, piRNA, circular RNA, and long non-coding RNA. Non-coding RNA participates in the regulation of almost every biological process, including both normal physiological activities and various disorders (Liu et al., 2017a; Sun et al., 2018; Ma et al., 2019; Ji et al., 2020). In this Research Topic, two different groups contributed two review papers summarizing the role of non-coding RNAs in colorectal cancer and gastric cancer, respectively. microRNA is among the most critical and intensively studied non-coding RNA molecule. microRNA has multiple roles in colorectal cancer (Liu et al., 2016; Liu et al., 2017b; Liu et al., 2018). The reprograming of cellular and systematic metabolism is a key hallmark of cancer (Liu and Gu, 2022a). Xiong et al. wrote a review about how microRNA is involved in the modulation of cancer metabolism and how we can develop novel treatment methods for colorectal cancer by targeting the microRNA-cancer metabolism pathways. Mitochondrion is a crucial organelle not only in cell metabolism, but also in cell fate decision (like in apoptosis and ferroptosis) (Liu and Gu, 2022b). In the other review by Chen et al., authors comprehensively described the functions of different types of non-coding RNAs in mediating mitochondria dynamics in gastric cancer. To begin with, they introduced the biogenesis and mechanism of action of several types of non-coding RNAs. Then they provided evidence that dysregulated mitochondria dynamics is associated with tumorigenesis. Next, they linked non-coding RNA and mitochondria dynamics to show the pathological relevance of non-coding RNA in gastric cancer by regulating mitochondria dynamics, which suggests promising targets in gastric cancer therapy.

In a metastatic colorectal cancer case, ELBassiouny revealed that a 70-year-old woman, who was diagnosed with right-sided CRC (T3/N1) with metastases, underwent cancer progression after first-line panitumumab and capecitabine, followed by second-line bevacizumab and oxaliplatin treatment. Then third-line treatment with trifluridine/tipiracil was given to her. After that, her disease was stabilized for more than 2 years. This is a good case showing the efficacy of trifluridine/tipiracil as a third-line drug for metastatic colorectal cancer. Additionally, Zhong et al. developed a nomogram and risk classification system, which is effective in predicting the cancer-specific survival of lymph-node- positive rectal cancer patient after radical proctectomy. This method may benefit the individualized postoperative survival prediction in these patients.

To summarize, we have incorporated six papers in either basic research or clinical practice in gastrointestinal cancers. We believe that this Research Topic will help the readers to get familiar with the latest advances in gastrointestinal cancer field.
